# Diseases of the Heart

**Published:** 1909-09

**Authors:** 


**Diseases of the Heart.** By JAMES MACKENZIE, M.D. Pp. xix, 386. London: Oxford Medical Publications. 1908.

This book is the outcome of Dr. Mackenzie's life-long experience of heart disease, gained by the use of new methods, together with the exercise of a peculiarly original mind. The author makes his stand upon certain facts; the heart is a muscular tube, its function is a peculiar form of peristalsis, and all forms of cardiac disease are to be regarded as interferences with the force and other qualities of this peristaltic contraction. The gravity of such diseases can be expressed in terms of the degree to which the reserve force of the myocardium is encroached upon. Disease of the heart should be studied from the point of view of its effect upon the physiology as well as on the anatomy of the heart. Throughout his book Dr. Mackenzie insists upon these facts, and supports his contentions by a wealth of data derived largely from graphic records of the venous pulse. These are most ingeniously interpreted, and as a rule the interpretations are convincing. In a few instances, however, the reader feels that while the author's theories cannot be gainsaid, they need support from evidence of other kinds before they meet with general and final acceptance.

Dr. Mackenzie's methods have perhaps done their best work in discovering to us the meaning of the various forms of arrhythmia ; and it is no exaggeration to say that his book was worth writing, if only for the sake of explaining his views as to the causation of these obscure disorders. But there is beyond this very much that is of the highest value ; the account of angina pectoris and allied phenomena, and the action of digitalis on the cardiac functions with the indications for its use, should be singled out for special mention. Dr. Mackenzie proves himself not only a builder of new ideas, but also an iconoclast of a very vigorous type. This is specially apparent in his attack on the Nauheim methods, " by which blood-pressure may be raised if it is too low, and lowered if it is too high." After all, it is almost an impertinence to criticise a book which embodies the findings of so vast an amount of diligent and original work, tempered by wholesome common sense. It is a classic, and will always mark a period of great advance in our knowledge of heart disease. Such a book should be read by every physician and general practitioner as a supplement to (not instead of) the more encyclopædic works on the same subject.

